# Airway Sequelae After Mechanical Ventilation for COVID-19: Protocol for a Scoping Review

**DOI:** 10.2196/41811

**Published:** 2023-11-29

**Authors:** Estephania Candelo, Oriana Arias-Valderrama, Jacobo Triviño-Arias, Felipe Quiroz, Daniel Francisco Isaza-Pierotti, William Victoria, Luis F Tintinago

**Affiliations:** 1 Head and Neck Department Fundacion Valle del Lili Cali Colombia; 2 Centro de Investigaciones Clinicas Fundacion Valle del Lili Cali Colombia; 3 Facultad de Ciencias de la Salud ICESI University Cali Colombia

**Keywords:** airway, sequelae, COVID-19, mechanical ventilation, SARS-CoV-2, scoping review, pulmonary, mortality, voice production, health care cost, health intervention

## Abstract

**Background:**

The epidemiology, morbidity, and burden of disease related to airway sequelae associated with invasive mechanical ventilation in the context of the COVID-19 pandemic remain unclear.

**Objective:**

This scoping review aims to summarize the current knowledge regarding airway sequelae after severe SARS-CoV-2 infection. This knowledge will help guide research endeavors and decision-making in clinical practice.

**Methods:**

This scoping review will include participants of all genders, and no particular age group who developed post–COVID-19 airway-related complication will be excluded. No exclusion criteria will be applied from country, language, or document type. The information source will include analytical observational studies. Unpublished data will not be completely covered as gray literature will be covered. A total of 2 independent reviewers will participate in the process of screening, selection, and data extraction, and the whole process will be performed blindly. Conflict between the reviewers will be solved through discussion and an additional reviewer. The results will be reported by using descriptive statistics, and information will be displayed on RedCap (Research Electronic Data Capture).

**Results:**

The literature search was conducted in May 2022 in the following databases: PubMed, Embase, SCOPUS, Cochrane Library, as well as LILACS and gray literature to identify observational studies; a total of 738 results were retrieved. The scoping review will be finished by March 2023.

**Conclusions:**

This scoping review will describe current knowledge on the most frequently encountered laryngeal or tracheal sequelae in patients exposed to mechanical ventilation due to SARS-CoV-2 infection. This scoping review will find the incidence of airway sequelae post COVID-19 and the most common sequelae such as airway granuloma, vocal fold paralysis, and airway stenoses. Future studies should evaluate the incidence of these disorders.

**International Registered Report Identifier (IRRID):**

DERR1-10.2196/41811

## Introduction

Airway sequelae can be due to several etiologies and cause disability by problems related to breathing, deglutition, and voice production [1-3]. Since the early decades of the twentieth century, when orotracheal intubation (OTI) became popular in clinical practice, the most frequent etiology of airway sequelae is iatrogenic and secondary to invasive mechanical ventilation (IMV) [1]. The duration of IMV not only positively correlates with the probability of developing airway sequelae but also with severity [2,3]. These sequelae include the need for alternative airway (ie, tracheostomy), and the development of cicatricial lesions. For instance, according to Johnson et al [4], in the United States for 2016, the nationwide estimated incidence of laryngotracheal stenosis at 45 days from discharge among patients exposed to IMV was 1.98 cases per 1000 discharges. Current knowledge on airway sequelae in SARS-CoV-2 infection survivors exposed to IMV is limited. A preliminary search of PROSPERO, MEDLINE, the Cochrane Database of Systematic Reviews, and Joanna Briggs Institute (JBI) Evidence Synthesis was conducted, and no current or in-progress scoping reviews or systematic reviews on the topic were identified.

The COVID-19 pandemic has imposed great challenges to humanity, representing the most important public health concern in recent times. Although most people recover from SARS-CoV-2 infection, many are left with sequelae [5]. Every COVID-19–related death could result in 5 quality-adjusted life-years lost, and at least 30% of the burden of the disease could be attributable to disability [6]. While the economic impact of adult laryngotracheal stenosis has been described [7], it remains unclear in the setting of the COVID-19 pandemic. A survey on 488 patients at day 60 after discharge from 38 hospitals in Michigan [8] found that 188 (38.5%) did not return to normal activities, 45 (9.2%) did not return to work due to health issues, 75 (15.4%) reported persistent cough, 193 (39.5%) reported dyspnea, 34 (7%) used continuous positive airway pressure, and 32 (6.6%) required supplementary oxygen. Although the authors did not discuss the possibility of airway sequelae in some of the surveyed patients, their findings may raise concerns about that possibility.

The pathophysiology of airway lesions associated with IMV is believed to involve processes ranging from the initial insult (ie, the exerted pressure by the endotracheal tube pressure on the respiratory mucosa, traumatic intubation, etc) causing tissue damage, through the mechanisms of repair (ie, inflammation, scarring, keloid formation, etc) [9]. Several risk factors for developing airway sequelae associated with IMV have been recognized [4,10-13], many related to pathophysiological processes. These can be categorized into sociodemographic characteristics, comorbidities, medications, and other variables related to the intubation-extubation process. Interestingly, there is some evidence that SAR-CoV-2 may have direct effects on the airway mucosa, such as cytopathic insult [14-17]. Perhaps, airway sequelae could develop in SARS-CoV-2 infection survivors exposed to noninvasive mechanical ventilation.

The proportion of patients with COVID-19 requiring IMV varies across different reports. In the HIFLO clinical trial, 18.7% of the 652 confirmed severe cases required IMV [18]. A study from Seattle, WA, reported that 75% of 24 patients with respiratory failure required IMV [19]. Other authors from China reported that 47.2% of 36 intensive care unit–admitted patients required IMV [20]. Another study from New York analyzed 2634 hospitalized patients, from which 12.2% required IMV [21]. One Italian study describes that 88% of 1300 intensive care unit patients required IMV [22]. Although the proportion of patients requiring IMV is not described, other Chinese researchers reported 44,415 cases, from which 14% were severe and 5% critical [23]. In a prospective cohort of 1357 confirmed patients with COVID-19 from Brazil [24], 421 (31%) required IMV, and from these, only 172 (40.9%) survived and were discharged. From those who survived, 95 (55.2%) were followed by video endoscopy at approximately day 100 after extubation; 38 of these patients presented laryngotracheal lesions, resulting in an incidence of 40%, with 6.3% of the cases being severe. The authors suggest that major risk factors for developing laryngotracheal lesions after surviving IMV for severe COVID-19 include larger endotracheal tubes, prone position, duration of intubation, increased leucocyte count, and coagulopathy as determined by elevated D-dimer or PT/INR [25,26]. In addition, widely used pronation maneuvers may increase the chance of laryngotracheal trauma as higher endotracheal pressure may be required, increasing the chances of airway trauma [27].

In patients with prolonged IMV (>96 h) [28], since prepandemic times, early tracheostomy (at days 7-14 after OTI) was widely accepted to be associated with better clinical outcomes, including lower complication rates, such as airway sequelae, among others [29]. However, COVID-19 guidelines recommend waiting for this procedure to avoid SARS-CoV-2 spread among health care workers, resulting in prolonged IMV up to 4 weeks [15]. In a preliminary report on clinical outcomes in 543 patients with post–COVID-19 tracheostomy from the United Kingdom, by November 2020, the number of days between OTI and tracheostomy ranged between 0 and 35 (median 16, IQR 13-22) [16]. Early on in the pandemic in Italy, the world was alerted to expect a surge in the incidence of airway sequelae because of the overwhelming number of critical patients who would be exposed to prolonged IMV [17].

The epidemiology, morbidity, characteristics, and burden of the disease related to airway sequelae associated with mechanical ventilation in the context of the COVID-19 pandemic remain unclear. The purpose of this study is to perform a scoping review based on a systematic literature review to summarize current knowledge regarding these conditions. Putting in perspective this knowledge will help guiding research endeavors and decision-making in clinical practice.

## Methods

The proposed scoping review will be conducted in accordance with the JBI methodology for scoping reviews [30].

### Review Questions

The research questions for this study are as follows:

- What is the current state of knowledge on airway sequelae in patients after mechanical ventilation for severe COVID-19?
- What is the epidemiology?
- What are the most common features of the lesions?
- What is the most common approach to treatment?
- What are the knowledge gaps and future insights?

### Eligibility Criteria

#### Participants

This review will consider studies of any design reporting airway sequelae encountered in patients of both sexes and all ages who survived after exposure to mechanical ventilation in the context of an infection with the SARS-CoV-2. Studies describing patients exposed to both invasive and noninvasive mechanical ventilation will be considered.

#### Inclusion Criteria

The following studies will be selected for this review:

- Studies including patients with airway sequelae after SARS-CoV-2 infection describing frequency, clinical features, and complications.
- Studies providing information about the effects and extent of complication in airways after mild, moderate, and severe SARS-CoV-2 infection.
- Studies providing information about the medical management of patients with airway sequelae after SARS-CoV-2 infection.

#### Exclusion Criteria

The exclusion criteria were as follows:

- Lack of full-text availability.
- Manuscripts describing an airway sequela before the COVID-19 pandemic.
- Review, state of the art article, comments, or expert opinions were not considered in this scoping review.
- Other SARS-CoV-2 infection sequelae, which do not include the airway, were not considered for the present scoping.

### Concept

Airway sequelae developed after exposure to mechanical ventilation as part of the treatment for SARS-CoV-2 infection. These conditions may include the need for an alternative airway (ie, trachestomy) and cicatricial lesions, such as fistulae, granulomas, synechiae, malaciae, and other causes of stenosis, among others. This scoping review will include patients with early airway intervention after discharge and minor surgical interventions to address early post-tracheostomy complications and long-term sequelae for this group of patients with and without intervention. Finally, the scoping review will not be limited by the time frame of the airway complication after the SARS-CoV-2 infection.

### Context

This review will consider studies that were published in the context of the COVID-19 pandemic. The scoping review would not be limited by language, but it will focus on the COVID-19 pandemic time frame from early 2020 to 2023.

### Types of Sources

The scoping review will include any source of scientific evidence. It may be quasi-experimental and observational in nature. It will not discriminate among quantitative, qualitative, and mixed methods study designs. In addition, systematic reviews as well as text and opinion papers will be considered for inclusion in the proposed scoping review.

The proposed scoping review will be conducted in accordance with the JBI methodology for scoping reviews [31] and in line with the Preferred Reporting Items for Systematic Reviews and Meta-Analyses extension for Scoping Reviews (PRISMA-ScR) [32].

### Search Strategy

The search strategy is intended to locate published scientific studies that meet the inclusion criteria. A preliminary search was performed by a librarian on May 17, 2022. An initial search of Ovid MEDLINE identified the following keywords and index terms: covid-19, SARS-CoV-2, long COVID, coronavirus disease 2019, post-acute covid, covid sequelae, sequelae, and airway. Using these keywords and index terms, a second search on Ovid MEDLINE, PubMed, Scopus (Elsevier), Embase, Cochrane Library, Web of Science, LILACS, and gray literature resulted in 738 article references. A detailed search strategy is provided in Multimedia Appendix 1.

The search strategy included all identified keywords and index terms. All the key terms were adapted for each included database and information source. Search terms will be created by the entire team of reviewers, though the search will be conducted by 1 person. The reference list of all included sources of evidence will be screened for additional studies. Studies published in any language will be included.

### Study or Source of Evidence Selection

The 738 article references were collated and uploaded to EndNote 20 (Clarivate Analytics), and 255 duplicates were removed. This resulted in a total of 483 references. Titles and abstracts will be screened by 2 independent researchers. Potentially relevant papers will be retrieved in full, and their citation details imported into the JBI System for the Unified Management, Assessment, and Review of Information. Articles published in languages different from English or Spanish will be initially translated through Google Translate, and, if deemed to meet the inclusion criteria, the full text will be professionally translated.

The full text of selected citations will be assessed in detail against the inclusion criteria by the same 2 independent researchers. Reasons for exclusion of full-text papers that do not meet the inclusion criteria will be recorded and reported in the scoping review. Any disagreements that arise between the researchers at each stage of the selection process will be resolved through discussion or with a third researcher. The results of the search will be reported in full in the final scoping review and presented in a PRISMA (Preferred Reporting Items for Systematic Reviews and Meta-Analyses) flow diagram [33].

### Data Extraction

Data will be extracted from papers included in the scoping review by 2 independent researchers using a spreadsheet to include the following variables: title, authors, year of publication, origin or country of origin (where the source was published or conducted), aims or purpose, population and sample size within the source of evidence (if applicable), methodology or methods, intervention type, comparator and their details (eg, duration of the intervention; if applicable), duration of the intervention (if applicable), outcomes and their details (eg, how measured; if applicable), and key findings that relate to the scoping review questions.

The draft spreadsheet will be modified and revised as necessary during the process of extracting data from each included paper. Modifications will be detailed in the full scoping review. Any disagreements that arise between the researchers will be resolved through discussion or with a third researcher. Authors of papers will be contacted to request missing or additional data, where required.

### Data Analysis and Presentation

A narrative description of the findings will be discussed in the framework of the review questions. The data will be summarized and presented in tables.

### Reference Searches

Citation tracking criteria will be used to identify important articles relevant to the topic of interest; this will also be done by using the reference list of a paper or citations of a paper by other articles to identify additional manuscripts relevant to the topic of the study, as it was previously mentioned by Diaz-Ordoñez et al [34]. Following that, papers from the list that have already been examined will be removed. If a paper meets the inclusion criteria, potential new manuscripts will be identified by checking the reference list of the included paper [35].

## Results

The search was performed on May 17, 2022, in MEDLINE, LILACS, the Cochrane Library, ScienceDirect, and OpenGrey; a total of 214 results were retrieved. Complete review of the manuscripts is expected in August 2022. All potential manuscripts will be imported into the reference management software Rayyan QCRI 23 [36]. The results of this scoping review will identify and describe the epidemiology, clinical characteristics, classification, and management of upper airway sequelae after SARS-CoV-2 infection.

## Discussion

Airway sequelae due to SARS-CoV-2 infection are described in most observational studies, most from the United States and Europe. The compromise of the upper airway was related to mechanisms to prevent the spread of the virus; as these interventions increased, the lesions became more severe. In the following years, the pressure over climate change increases the cross-species transmission of their associated viruses an estimated 4000 times [37], which may increase the chance of pandemics such as COVID-19. For this reason, all the lessons learned about it should change how the following epidemics will be addressed. For example, developing more efficient strategies with the help of technology or artificial intelligence to contain the respiratory virus would decrease the sequelae among patients and the related cost.

At the beginning of the pandemic, interventions began not only thinking about the well-being and recovery of patients but also about avoiding the transmission of the virus to health personnel. However, the involvement of the upper airway was related to the mechanisms used to prevent the spread of the virus [38]; as these interventions increased, injuries became more prevalent and more severe [17]. These sequelae are complications that, given the high need to perform the causal procedures, are a problem that will increase in the coming years. Otorhinolaryngologists and head and neck surgeons should know them in depth to make a diagnosis and timely treatment to reduce the high disease burden of affected patients evidenced in our review. Airway sequelae due to SARS-CoV-2 infection are described in most observational studies, most from the United States and Europe. The compromise of the upper airway was related to mechanisms to prevent the spread of the virus; as these interventions increased, the lesions became more severe.

Some of the frequent airway sequelae documented in the literature include the development of tracheal and laryngotracheal stenosis after prolonged intubation and subsequent tracheostomy [14,39,40]. Other articles reported aerodigestive complications, which include vocal fold paralysis and paresis, muscle tension dysfunction, laryngotracheal hypersensitivity, and laryngopharyngeal reflux [41]. Allisan-Arrighi et al [41] reported a broad spectrum of respiratory, swallowing, and voice difficulties, which cause distress in patients recovering from COVID-19 infection. The sequelae included structural and anatomic inflammatory changes of the upper airway trachea with the following frequency: 16.13% for tracheal stenosis, 16.13% arytenoid ankylosis, and 16.13% posterior subglottic stenosis [41]. In other articles, authors report airway granulomas as a frequent complication of severe COVID-19 infection [42]. In addition, Guarnieri et al [43] reported a high incidence of tracheomalacia after tracheostomy in patients with severe SARS-CoV-2 infection, as the tracheostomized patient had a longer intensive care unit and hospital stay [43]. In their study, Guarnieri et al [43] showed a 5% incidence of tracheomalacia, which is 10 times higher than the reported literature [43].

In the following years, the environmental and physiological stress to which the animals are subjected translates into an alteration in their immune system that provides greater susceptibility not only to being hosts of the microorganism but also to promoting its replication and therefore increasing the risk of a zoonotic overflow [44]. Environmental issues may be associated with pandemic occurrence [44]. For this reason, all the lessons learned in this regard should change how the following epidemics will be addressed; for example, by developing more efficient strategies [45] and artificial intelligence to contain the respiratory virus. These strategies would decrease the sequelae between patients and the high cost. Patients with pre-existing airway disease are thought to be at a higher risk of developing severe outcomes from acute COVID-19 [46]. Most of the evidence reports these effects on lower airways, and the effects on upper airways may start to be reported in the next few years.

This study has several limitations, including the following: a summary of results that would not reflect the entirety of the literature available needs to be included. Additionally, the scoping review does not consider the risk of bias assessment [47]. For that reason, some included studies may incur bias. In our case, the data synthesis needs to address the weight of the evidence. We performed a narrative summary of the results and a descriptive form of the quantitative data. Finally, the broad search would dilute some specific results essential to the main objective of the scoping review.

## Appendix

Multimedia Appendix 1 Search strategy.

## Abbreviations

IMV: invasive mechanical ventilation
JBI: Joanna Briggs Institute
OTI: orotracheal intubation
PRISMA: Preferred Reporting Items for Systematic Reviews and Meta-Analyses
PRISMA-ScR: Preferred Reporting Items for Systematic Reviews and Meta-Analyses extension for Scoping Reviews
RedCap: Research Electronic Data Capture
