# Supplementary material for: Airway Sequelae After Mechanical Ventilation for COVID-19: Protocol for a Scoping Review
Source: JMIR Res Protoc. 2023 Nov 29;12:e41811. doi: 10.2196/41811 (PMC10719820; doi:10.2196/41811)
Supplement: Multimedia Appendix 1 [file resprot_v12i1e41811_app1.docx]

# Multimedia Appendix 1. Search strategy.

| **#** | **Searches** | **Results** |
| --- | --- | --- |
|  | **Ovid MEDLINE including (PubMed and EMBASE)** |  |
| 1 | exp covid-19/ or exp SARS-CoV-2/ or exp long COVID/ or exp coronavirus disease 2019/ | 393290 |
| 2 | (covid-19 or covid or SARS-CoV-2 or long covid or post-acute covid or covid sequelae).ti,ab. | 501188 |
| 3 | 1 or 2 | 539000 |
| 4 | post-acute.ti,ab. or long.ti,ab. or sequelae.ti,ab. | 4145956 |
| 5 | 3 and 4 | 33857 |
| 6 | airway.ti,ab. | 397854 |
| 7 | 5 and 6 | 313 |
| 8 | remove duplicates from 7  EBM Reviews - Cochrane Central Register of Controlled Trials <April 2022> 9  EBM Reviews - Cochrane Database of Systematic Reviews <2005 to May 11, 2022> 1  Embase <1974 to 2022 May 16> 88  Ovid MEDLINE(R) ALL <1946 to May 13, 2022> 116 | **214** |
|  | **Scopus (Elsevier)** |  |
| 1 | ((covid-19 OR covid OR SARS-CoV-2 OR “long covid” OR “post-acute covid” OR “covid sequelae”) AND (long OR post-acute OR sequelae)) AND (airway) | **311** |
|  | **Cochrane Library** |  |
|  | **(((covid-19 OR covid OR SARS-CoV-2 OR “long covid” OR “post-acute covid” OR “covid sequelae”) AND (long OR post-acute OR sequelae)) AND (airway)):ti,ab,kw** | **0** |
|  | **LILACS** |  |
|  | **(COVID19) OR (SARS-CoV2) AND (enfermedad severa) OR (post COVID) AND (secuelas) AND (via aerea )** | **0** |
|  | **Web of Science** |  |
| 1 | ((covid-19 OR covid OR SARS-CoV-2 OR “long covid” OR “post-acute covid” OR “covid sequelae”) AND (long OR post-acute OR sequelae)) AND (airway) | **213** |

738 total article references

255 duplicates found in EndNote

**483 total references in EndNote**
